# A meta-analysis of the efficacy of fibromyalgia treatment according to level of care

**DOI:** 10.1186/ar2455

**Published:** 2008-07-15

**Authors:** Javier Garcia-Campayo, Jesus Magdalena, Rosa Magallón, Esther Fernández-García, Montserrat Salas, Eva Andrés

**Affiliations:** 1 Miguel Servet Hospital, University of Zaragoza, Zaragoza, Spain; 2 Letux Health Centre, Letux, Zaragoza, Spain; 3 Arrabal Health Centre, Zaragoza, Spain; 4 Miguel Servet Hospital, University of Zaragoza, Zaragoza, Spain; 5 Government of Aragon, Zaragoza, Spain; 6 University of Zaragoza, Zaragoza, Spain; 7 Grupo Aragonés de Investigación en Atención Primaria, Red de Actividades Preventivas y de Promoción de la Salud (REDIAPP) (G06/128), Instituto Aragonés de Ciencias de la Salud (IACS), Zaragoza, Spain

## Abstract

**Introduction:**

The aim of this paper was to compare the efficacy of the treatments for fibromyalgia currently available in both primary care and specialised settings.

**Methods:**

Published reports of randomised controlled trials (RCTs) researching pharmacological and non-pharmacological treatments in patients with fibromyalgia were found in the MEDLINE, EMBASE, the Cochrane Central Register of Controlled Trials and PsychInfo databases. The most recent electronic search was undertaken in June 2006.

**Results:**

We identified a total of 594 articles. Based on titles and abstracts, 102 full articles were retrieved, 33 of which met the inclusion criteria. These RCTs assessed 120 treatment interventions in 7789 patients diagnosed with primary fibromyalgia. Of them, 4505 (57.8%) were included in the primary care group of our study and 3284 (42.2%) in the specialised intervention group. The sample was mostly made up of middle-aged women, who have had fibromyalgia for a mean period of 6 to 10 years. The mean effect size of the efficacy of the 120 treatment interventions in patients with fibromyalgia compared with controls was 0.49 (95% confidence interval [CI] = 0.39 to 0.58; p < 0.001). In the primary care group it was 0.46 (95% CI = 0.33 to 0.58) while in specialised care it was 0.53 (95% CI = 0.38 to 0.69), with no statistical significance in the differences. We analysed the efficacy of treatments by comparing primary and specialised care in the different fibromyalgia groups and there were no significant differences. The variables of the studies that affected the improvements in the efficacy of fibromyalgia treatment were low quality of the studies and a shorter duration of treatment. However, both factors were biased by the heterogeneity of the studies. Other variables that also improved outcome and were not biased by the heterogeneity of the studies, were younger age of the patients and shorter duration of the disorder. On the contrary, gender and type of treatment (pharmacological vs. psychological) did not affect outcome.

**Conclusion:**

Based on this meta-analysis and despite the heterogeneity of specialised care studies and of the other limitations described in this article, treating fibromyalgia in specialised care offers no clear advantages.

## Introduction

Fibromyalgia is a chronic musculoskeletal pain disorder of unknown aetiology, characterised by widespread pain and muscle tenderness and often accompanied by fatigue, sleep disturbance and depressed mood [1,2]. With an estimated lifetime prevalence of approximately 2% in community samples [3], it accounts for 15% of outpatient rheumatology visits and 5% of primary care visits [4]. The prognosis for symptomatic recovery is generally poor [5]. A wide variety of interventions are used in the management of this disorder, although there is no clear consensus on the treatment of choice and fibromyalgia remains relatively refractory to treatment.

A number of meta-analyses and reviews have been conducted on the pharmacological [6-8] and non-pharmacological [9,10] treatments available for fibromyalgia. The studies main objectives are to guide clinicians in their everyday practice using evidence-based decisions. However, the aim of our current study is rather different. The high prevalence and clinical impact of fibromyalgia makes it a significant public health problem given its high cost. In Spain and other public health systems, a difficult cost-benefit decision must be taken as to which level of the health care system these patients should be treated in: either in specialised settings, which many patients prefer, or in primary care, which is usually more cost-effective. To our knowledge, there is no published meta-analysis on this subject.

We carried out a systematic review and meta-analysis of all randomised controlled trials (RCTs) of pharmacological and non-pharmacological treatments that are available in standard primary care settings and those that are administered in standard secondary care settings of public health care systems in developed countries for the treatment of fibromyalgia. The aim of this paper is to compare the efficacy of the treatments for fibromyalgia available in both settings using the most important outcomes assessed in this disorder, such as pain, quality of life, depression, etc.

## Materials and methods

We followed the QUOROM guidelines for reporting meta-analyses [11].

### Database search

Published reports of RCTs researching pharmacological or non-pharmacological treatments in patients with fibromyalgia were found in the following databases: MEDLINE (1966–2006), EMBASE (1988–2006), The Cochrane Central Register of Controlled Trials (the Cochrane Library Issue 2006) and Psychinfo (1987–2006). Search strategy is summarised in the additional data file. The search was performed without language restrictions but was limited to RCTs in humans. The last electronic search was undertaken in June 2006. All primary and review articles, as well as their references, were reviewed independently in duplicate. The authors of the original reports were contacted for additional information where needed.

### Selection criteria

Studies were screened for inclusion, by reviewing the title and published abstract, based on the following criteria:

#### Type of participants

The studies evaluated the treatment or management of fibromyalgia as indicated by the use of recognised diagnostic criteria, such as American College of Rheumatology (ACR) [1]. Despite the concept of primary fibromyalgia (patients in which fibromyalgia can not be explained by other medical disorders) not being accepted by the ACR, most studies on fibromyalgia, and many of the papers included in the meta-analysis, do accept this distinction. Therefore, it has been maintained to increase comparability.

#### Types of studies

The papers described a randomisation of treatment, placebo control and at least one group receiving an active (pharmacological or non-pharmacological) treatment.

#### Types of interventions

Treatment can be defined as pharmacological or non-pharmacological, and can be allocated to primary or specialised care. The duration of treatment was at least eight weeks.

#### Types of outcomes

Outcomes had to be measurable. One of the major problems in fibromyalgia is the wide variety of outcomes. Seven types of outcomes were included: pain, fatigue, quality of life, global function, anxiety/depression, insomnia and tender points. Each of them were assessed with several questionnaires.

**Figure 1:**
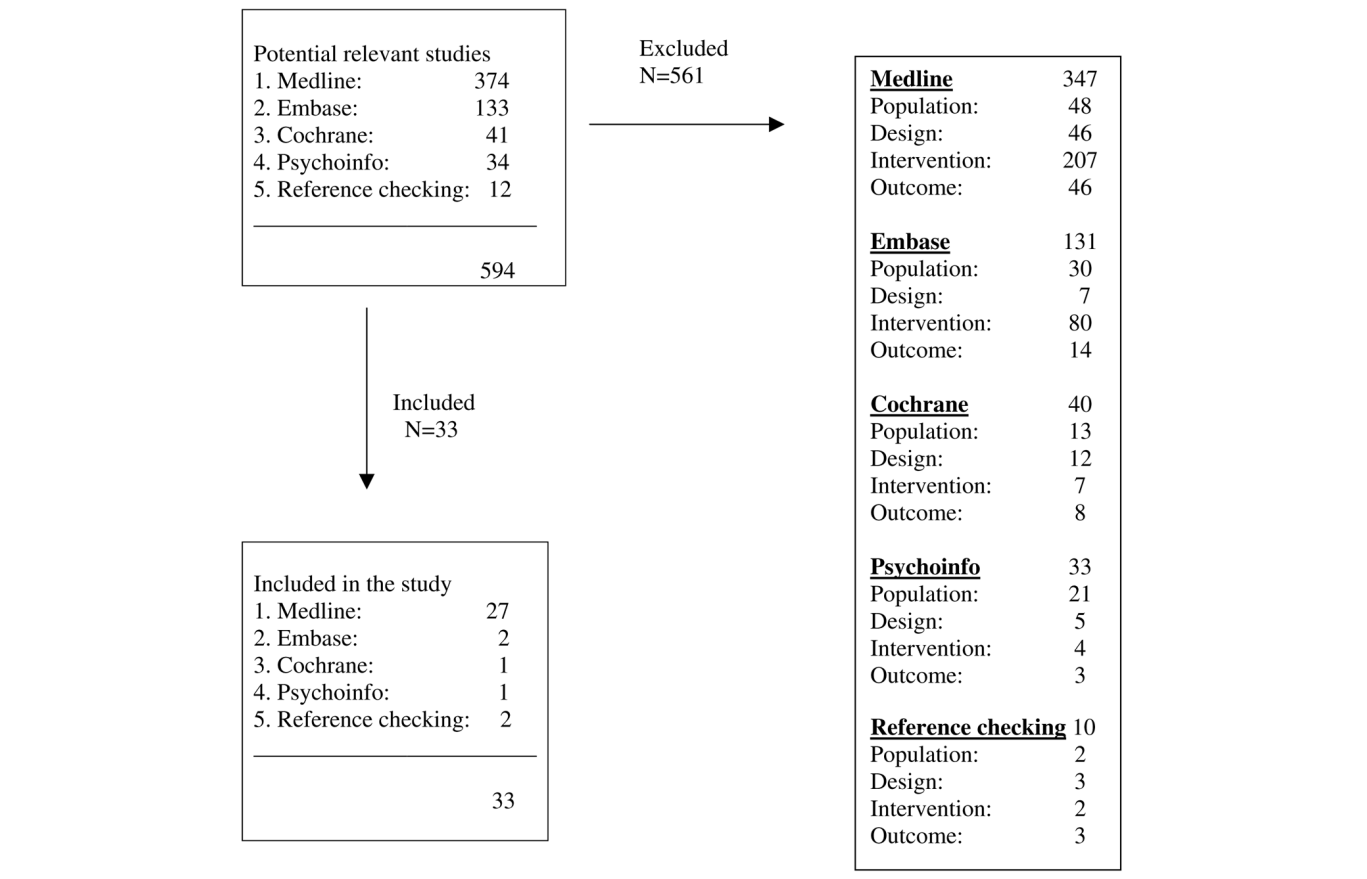
Flowchart showing the process of study selection.

Each study was reviewed in duplicate (by EF and JGC) for inclusion with substantial inter-rater agreement (kappa = 0.7). Disagreements were resolved by a consensus agreement. Reviews and abstracts were not considered. The study selection process flowchart is summarised in Figure 1.

### Allocation

All studies included were allocated to a level of health care (primary care or specialised care) and category of treatment (pharmacological or non-pharmacological) by a consensus with substantial inter-rater agreement (kappa = 0.91) from a panel of two general practitioners (RM and JM), a psychiatrist (JGC) and a psychologist (EF). A treatment was considered to be available at the primary care level when most general practitioners from most Western national health services were able to provide that treatment without any specific training. Tables 1 and 2 summarise which treatments were allocated to the primary and specialised care groups and to the pharmacological and non-pharmacological treatment groups. We have not included RCTs on acupuncture because of the recent meta-analyses showing that this treatment is not effective [10].

**Table 1:** Allocation of treatments according to level of care

**Primary care**	**Secondary care**
Amitriptyline	Pirlindole
Tramadol	Tropisetron
Milnacipran	Dehydroepiandrosterone (DHEA)
Moclobemide	Pramipexole
Fluoxetine	Malic acid
Cyclobenzaprine	Rehabilitation
Nortriptyline	Laser treatment
Duloxetine	Hyperbaric oxygen therapy
Pregabaline	Bright light treatment
Zolpidem	Aerobic exercise
	Exercise
	Stress-reduction treatment
	Chiropractic management
	Cognitive behavioural therapy
	Cognitive educational therapy
	Education training
	Behavioural insomnia therapy
	Music vibration

**Table 2:** Pharmacological and non-pharmacological treatments

**Pharmacological treatments**	**Non-pharmacological treatments**
Amitriptyline	Hyperbaric oxygen therapy
Cyclobenzaprine	Bright light treatment
Dehydroepiandrosterone (DHEA)	Aerobic exercise
Duloxetine	Education training
Fluoxetine	Behavioural therapy
Malic acid	Cognitive behavioural therapy
Milnacipran	Cognitive educational therapy
Moclobemide	Exercise
Nortriptyline	Rehabilitation
Pirlindole	Music vibration
Pramipexole	Chiropractic management
Pregabaline	Stress-reduction treatment
Tramadol	Behavioural insomnia therapy
Tropisetron	Laser
Zolpidem	

### Validity assessment

All included reports were then independently read by two reviewers (EF and JGC) who assessed the validity of the studies using the modified Oxford Scale (Table 3) [12,13]. The minimum score of an included trial was one and the maximum was six. Discrepancies were resolved by discussion or by consulting a third reviewer (RM).

**Table 3:** Modified Oxford Scale. Validity score (0 to 7)

Randomisation	
0 None	
1 Mentioned	
2 Described and adequate	
	
Concealment of allocation	
0 None	
1 Yes	
	
Double blinding	
0 None	
1 Mentioned	
2 Described and adequate	
	
Flow of patients	
0 None	
1 Described but incomplete	
2 Described and adequate	

### Data abstraction

A data abstraction form was created and the following data were included: number of patients and controls, gender (percentage of women), age (median), diagnosis, time of evolution of the disorder (years), severity of the disorder, level of health care (primary care or specialised), kind of treatment (pharmacological or non pharmacological), duration of treatment, modified Oxford Scale ratings and outcomes (ratings in different used scales of quality of life, pain, depression, anxiety, etc).

### Meta-analyses

Both dichotomous and continuous data were extracted. Continuous data were analysed as standardised differences in the means (SDM) with 95% confidence intervals (CI). Where mean values and standard deviations were not reported, the authors of the studies were contacted. If they did not reply and the data were presented graphically, data were extracted from the graphs. If this was not possible, the data were not considered. A random effects model was used by default. Analyses were performed using Comprehensive Meta-analysis, version 2 (Biostat, Englewood, NJ, USA). Data were graphically plotted using forest plots to evaluate treatment effects. Clinical heterogeneity was minimised using stringent diagnostic criteria for fibromyalgia and homogeneous criteria for the treatments and outcomes of the studies included in the meta-analysis.

## Results

### Literature search and study selection

We first performed our literature search in MEDLINE (374 hits), followed by EMBASE (133 hits), and subsequently in the Cochrane Library (41 hits) and in Psychinfo (34 hits). By checking references, we identified an additional 12 hits, resulting in a total of 594 articles (Figure 1). Based on titles and abstracts, 102 full articles were retrieved, 33 of which met the inclusion criteria [14-46]. These 33 studies are summarised in Table 4.

**Table 4:** Characteristics of the 33 selected randomised controlled trials and the patients studied in them

N	**Randomized controlled trial**	**Year**	**Country**	**Treatment**	**Level of care**	**% of women**	**Mean age**	**Length of treatment (years)**	**Simple size**	**Oxford scoring (quality)**	**Duration of disease at baseline (years)**	**Instruments used (outcome assessed)**
1	Carette	94	Canada	Amitryptiline Cyclobenzaprine	Primary care	93.8	44.4	24	208	3	7.7	Mc Gill-BPI (p) SIP (gf)
2	Russell	94	USA	Malic acid	Specialised care	90	49.5	8	24	3		VAS (p) TPI (tp)
3	Wolfe	94	USA	Fluoxetine	Primary care	100	50.4	3	42	5	13	TPI (tp) BDI (ad)
4	Carette	95	Canada	Amitryptiline	Primary care	95.5	43.8	8	22	2	6.9	VAS (p) VAS (i) VAS (gf)
5	Chesky	95	USA	Music vibration	Specialised care	92.6	48.8	30 minutes	26	3	11	VAS (p) TPI (tp)
6	Goldenberg	96	USA	Fluoxetine Amitryptiline	Primary care	90.3	43	6	31	5	5.7	VAS (p) FIQ (GF) BDI (ad) VAS (i) VAS (gf) VAS (f) TPI (tp)
7	Ginsberg	96	Belgium	Amitryptiline	Primary care	82.5	46	8	46	2	32	VAS (p) VAS (i) TPI (tp) VAS (f) VAS (gf) NTP (tp)
8	Moldofsky	96	Canada	Zolpidem	Primary care	95	42	2.5	19	4		NTP (tp) PGI (i)
9	Vlayen	96	Holland	Cognitive behavioural therapy Education training	Specialised care	87	44	6	131	5	10	BDI (ad)
10	Wigers	96	Norway	Aerobic exercise Stress-reduction treatment	Specialised care	92	44	14	48	3	10	VAS (p) VAS (i) VAS (f)
11	Pearl	96	Canada	Bright light treatment	Specialised care	100	38	10	14	2	5	VAS (p) VAS (f) VAS (i)
12	Kelli	97	Canada	Chiropractic treatment	Specialised care	-	49	4	19	4	8	VAS (p) NTP (tp)
13	Hannonen	98	Finland	Moclobemide Amitryptiline	Primary care	100	49	12	130	5	11.2	NTP (tp) VAS (p) VAS (f) VAS (i)
14	Yavuzer	98	Turkey	Moclobemide	Primary care	58	33	6	60	1		TPI (tp)
15	Ginsberg	98	Belgium	Pirlindole	Specialised care	85	40	4	61	4	2.9	VAS (p) TPI (tp) VAS (gf)
16	Russell	00	USA	Tramadol	Primary care	94	49	6	69	4	4.7	VAS (p) FIQ (gf) NTP (tp)
17	Heymann	01	Brazil	Amitryptiline Nortryptiline	Primary care	100	50	8	118	4		FIQ (gf) NTP (tp)
18	Färber	01	Germany	Tropisetron	Specialised care	92	48	1.5	403	3	11	Vas (p) NTP (tp)
19	Gowans	01	Canada	Exercise	Specialised care	88	47	23	50	3	9	FIQ (gf) BDI (ad) STAI (ad) NTP (tp)
20	Mannerkorpi	01	Sweden	Education training	Specialised care	100	46	24	58	4	8.7	FIQ (gf) QOLS (ql)
21	Gür	02	Turkey	Laser Amitryptiline	Primary care (Amytriptiline) – Specialised care (laser)	80	30	8	75	3	4.6	HADS (ad) FIQ (gf)
22	Joaquim	02	Sweden	Education training Behavioural therapy	Specialised care	100	45	12	53	6	3.6 pain	FIQ (gf) Mc Gill (p)
23	King	02	Canada	Exercise Education training	Specialised care	100	46	12	152	4		FIQ (gf) NTP (tp)
24	Lemstra	05	Canada	Rehabilitation	Specialised care	84.5	49.5	6	79	3	10	VAS (p) BDI (ad)
25	Schachter	03	Canada	Aerobic exercise (long-term and short-term)	Specialised care	100	42	16	143	4	3.5	VAS (p) FIQ (gf)
26	Arnold	04	USA	Duloxetine	Primary care	88	49	12	207	6	8.9	BDI (ad) BPI (ad) NTP (tp) CGI (gf) FIQ (gf)
27	Yildiz	04	Turkey	Hyperbaric oxigen therapy	Specialised care	70	40	2.5	50	2	4.5	VAS (p) NTP (tp)
28	Crofford	05	USA	Pregabaline	Primary care	92	48.5	8	529	5	9	VAS (p) MAF (f)
29	Arnold	05	USA	Duloxetine	Primary care	100	50	12	354	5		BPI (ad)
30	Gendreau	05	USA	Milnacipran	Primary care	98	47	12	125	6	4.1	FIQ (gf)
31	Finckh	05	Switzerland	Dehydroepiandrosterone (DHA)	Specialised care	100	59	12	52	6	13	HADS (ad) VAS (f)
32	Holman	05	USA	Pramipexole	Specialised care	94.4	48.5	14	60	6	8.4	BDI (ad) HAMD (ad) TPI (tp) FIQ (gf)
33	Edinger	05	USA	Cognitive behavioural therapy Sleep hygiene	Specialised care	100	49	6	47	3		Mc Gill (p) BPI (ad) SF-36 (ql)

Outcome types: P, Pain; QL, Quality of life; AD, Anxiety-depression; I, Insomnia; TP, Tender points; F, Fatigue; GF, Global Function.

Questionnaires: Mc Gill PQ, Mc Gill Pain Questionnaire; BPI, Brief Pain Inventory; VAS, Visual Analogue Scale; QOLS, Quality of Life Scale; BDI, Beck Depression Inventory; HADS, Hospital Anxiety and Depression Scale; HDS, Hamilton Depression Scale; STAI, State-trait Anxiety Inventory; PGI, Patient Global Impression; TPI, Tender Points Index; MAF, Multi-dimensional Assessment of Fatigue; FIQ, Fibromyalgia Impact Questionnaire; CGIS, Clinical Global Impression of Severity; SIP, Sickness Impact Profile.

Of the 69 studies that were excluded 23 were not an RCT; the patient population of 28 were not primary fibromyalgia (but secondary fibromyalgia or allied conditions) or the fibromyalgia criteria used were not ACR criteria [1]; in 16 studies the intervention had a duration shorter than eight weeks or it was so specific that it was not available in standard Western health care systems [47]; and two studies did not use comparable outcome measures. There was seven types of outcomes used in the studies selected from 16 questionnaires or tests summarised in Table 5.

**Table 5:** Questionnaires and outcome types used in the studies selected

Type of outcome	Questionnaires
Pain (p)	McGill Pain Questionnaire [48]
	Brief Pain Inventory [49]
	Visual Analogue Scale [50]
Quality of life (ql)	SF-36 [51]
	Quality of Life Scale (QOLS) [52]
Anxiety and depression (ad)	Beck Depression Inventory [53]
	Hospital Anxiety and Depression Scale [54]
	Hamilton Depression Scale [55]
	State-trait Anxiety Inventory [56]
Insomnia (i)	Visual Analog Scale [50]
	Patient Global Impression [57]
Tender points (tp)	Tender Points Index [58]
	Number of Tender Points according to American College of Rheumatology criteria [1]
Fatigue (f)	Visual Analog Scale [50]
	Multi-dimensional Assessment of Fatigue [59]
Global Function (gf)	Visual Analog Scale [50]
	Fibromyalgia Impact Questionnaire [60]
	Clinical Global Impression of Severity [57]
	Sickness Impact Profile (SIP) [61]

### Methodological quality of the included studies

Only 11 of the 33 included studies (33.3%) showed a rating of 5+ on the modified Oxford Scale, that is, a score of high methodological quality. Most of them (nine of 11) were pharmacological studies and the remaining two studies were psychological interventions. Many of them were recent studies, carried out in 2004 and 2005 (six of 11), as can be seen in Tables 4 and 6. The most commonly absent items were an adequate description of the flow of patients and adequate description of double blinding.

**Table 6:** Characteristics of the patients included in the meta-analysis

	**Total**	%	**Control group**	%	**Intervention group**	%
**Level of care**						
Primary care	4505	57.8	2127	57.6	2378	58.1
Specialised care	3284	42.2	1567	42.4	1717	41.9
Overall	7789	100.0	3694	100.0	4095	100.0
						
**Kind of treatment**						
Pharmacological	5706	73.3	2684	72.7	3022	73.8
Non-pharmacological	2083	26.7	1010	27.3	1073	26.2
Overall	7789	100.0	3694	100.0	4095	100.0
						
**Outcome assessed**						
Anxiety/depression	1195	15.3	570	15.4	625	15.3
Quality of life	115	1.5	51	1.4	64	1.6
Pain	2074	26.6	980	26.5	1094	26.7
Fatigue	650	8.3	320	8.7	330	8.1
Tender points	1533	19.7	738	20.0	795	19.4
Insomnia	397	5.1	196	5.3	201	4.9
Global function	1825	23.4	839	22.7	986	24.1
Overall	7789	100.0	3694	100.0	4095	100.0
						
**Methodological quality**						
1 to 2	595	7.6	289	7.8	306	7.5
3 to 4	3396	43.6	1577	42.7	1819	44.4
5 to 6	3798	48.8	1828	49.5	1970	48.1
Overall	7789	100.0	3694	100.0	4095	100.0
						
**Length of treatment (weeks)**						
0 to 8	3644	46.8	1750	47.4	1894	46.3
09 to 16	3467	44.5	1678	45.4	1789	43.7
17 to 24	678	8.7	266	7.2	412	10.1
Overall	7789	100.0	3694	100.0	4095	100.0
						
**Age**						
30 to 39	253	3.2	125	3.4	128	3.1
40 to 49	6662	85.5	3140	85.0	3522	86.0
50 to 59	874	11.2	429	11.6	445	10.9
Overall	7789	100.0	3694	100.0	4095	100.0
						
**Percentage of women**						
< 80	151	1.9	73	2.0	78	1.9
80 to 89	2258	29.0	1111	30.1	1147	28.0
90 to 99	2874	36.9	1297	35.1	1577	38.5
100	2506	32.2	1213	32.8	1293	31.6
Overall	7789	100.0	3694	100.0	4095	100.0
						
**Duration of the disorder (years)**						
0 to 5	1459	25.3	710	26.3	749	24.5
6 to 10	2987	51.9	1344	49.7	1643	53.8
11 to 15	1310	22.8	649	24.0	661	21.7
Overall	5756	100.0	2703	100.0	3053	100.0
						
**Country**						
Germany	410	5.3	206	5.6	204	5.0
Belgium	459	5.9	216	5.8	243	5.9
Brasil	278	3.6	132	3.6	146	3.6
Canada	1655	21.2	737	20.0	918	22.4
Finland	488	6.3	240	6.5	248	6.1
Holland	174	2.2	86	2.3	88	2.1
Norway	195	2.5	102	2.8	93	2.3
Sweden	254	3.3	126	3.4	128	3.1
Switzerland	276	3.5	135	3.7	141	3.4
Turkey	298	3.8	148	4.0	150	3.7
USA	3302	42.4	1566	42.4	1736	42.4
Overall	7789	100.0	3694	100.0	4095	100.0
						
**Year of publication**						
1994	620	8.0	234	6.3	386	9.4
1995	178	2.3	86	2.3	92	2,.2
1996	1306	16.8	637	17.2	669	16.3
1997	38	0.5	18	0.5	20	0.5
1998	724	9.3	349	9.4	375	9.2
2000	207	2.7	102	2.8	105	2.6
2001	926	11.9	460	12.5	466	11.4
2002	780	10.0	372	10.1	408	10.0
2003	392	5.0	196	5.3	196	4.8
2004	1241	15.9	621	16.8	620	15.1
2005	1377	17.7	619	16.8	758	18.5
Overall	7789	100.0	3694	100.0	4095	100.0

### Study characteristics

The review selected 33 RCTs that assessed 120 treatment interventions on 7789 patients diagnosed with primary fibromyalgia according to ACR criteria [1]. Of these, 4505 (57.8%) were included in the primary care group and 3284 (42.2%) in the specialised intervention group. The characteristics of the patients included in these studies are summarised in Table 6. The sample was made up of middle-aged women, who had the disorder for between six and 10 years (51.9%), treated mainly with pharmacological approaches (73.3%). The outcome types most frequently assessed were pain (26.6%) and global function (23.4%). Most of the patients were from studies carried out in the USA and Canada (63.6%) and were published after 2000 (61.5%). There were no significant differences in any of the variables studied between control and intervention groups.

The mean effect size of the efficacy of the 120 treatment interventions on patients with fibromyalgia compared with efficacy in controls, regardless of the outcome type assessed or the questionnaire used, was 0.49 (95%CI = 0.39 to 0.58; p < 0.001). This is a medium size effect [62], but it is significant. When we compared the efficacy of these treatments on fibromyalgia, after allocating the treatments to primary or specialised level of care, regardless of the type of outcome assessed or the questionnaire used, mean effect size of efficacy in primary care was 0.46 (95%CI = 0.33 to 0.58) while in specialised care was 0.53 (95%CI = 0.38 to 0.69. These differences are not significant.

When we analysed the efficacy of treatments on the different fibromyalgia outcome types (Table 7), we observed that there is an overlapping of the interval scores comparing primary and specialised care for all outcome types. This means that there are no significant differences. There are insignificant differences favouring secondary or specialised care for tender points (mean = 0.28; 95% CI = 0.12 to 0.68 for primary care; mean = 0.50, 95% CI = 0.0 to 1.0 for specialised care) and pain (mean = 0.48, 95% CI = 0.30 to 0.66 for primary care; mean = 0.73, 95% CI = 0.41 to 1.05 for specialised care). On the other hand, there are insignificant differences in favour of primary care for insomnia (mean = 0.57, 95% CI = 0.15 to 0.99 for primary care; mean = -0.18, 95% CI = -0.62 to 0.27 for specialised care), anxiety/depression (mean = 0.59, 95% CI = 0.10 to 1.08 for primary care; mean = 0.40, 95% CI = 0.12 to 0.67 for specialised care), and fatigue (mean = 0.30, 95% CI = 0.05 to 0.56 for primary care; mean = 0.22, 95% CI = -0.08 to 0.52 for specialised care). For specialized care, there are minimal differences also nonsignificant (surely the cause is higher heterogeneity in these studies). Global function, thought to capture the whole impact of the disease, was quite similar in both levels of care (0.53 in primary care; 0.54 in specialised care). The quality of life outcome could not be compared because there were no studies in primary care assessing this variable.

**Table 7:** Efficacy of treatments by fibromyalgia outcomes according to level of care

**Global function**		**Pain**			**Tender points**			**Quality of life**			**Anxiety/depression**			**Insomnia**			**Fatigue**		
Low-Up Limit		Std dif in means	Low-Up Limit		Std dif in means	Low-Up Limit		Std dif in means	Low-Up Limit		Std dif in means	Low-Up Limit		Std dif in means	Low-Up Limit		Std dif in means	Low-Up Limit	

0.30	0.76	**0.48**	0.30	0.66	**0.28**	-0.12	0.68				**0.59**	0.10	1.08	**0.57**	0.15	0.99	**0.30**	0.05	0.56
0.32	0.77	**0.73**	0.41	1.05	**0.50**	0.00	1.00	**1.22**	0.49	1.95	**0.40**	0.12	0.67	**-0.18**	-0.62	0.27	**0.22**	-0.08	0.52
0.38	0.69	**0.54**	0.38	0.70	**0.37**	0.05	0.68	**1.22**	0.49	1.95	**0.44**	0.20	##	**0.22**	-0.09	0.52	**0.27**	0.07	0.46

**Figure 2:**
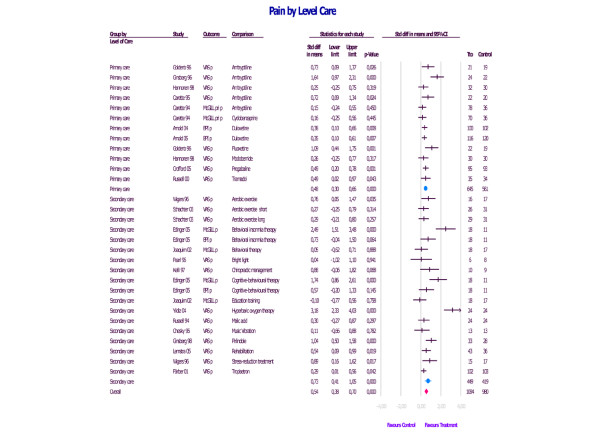
Efficacy of the treatments allocated to both levels of care according to the type of pain in patients with fibromyalgia.

As an example, we have included the efficacy of the treatments allocated to both levels of care in the outcome of pain in patients with fibromyalgia (Figure 2). This outcome is one of the most important in this disorder and the most thoroughly assessed in the studies reviewed. We can observe that there are insignificant differences favouring specialised care (0.73 for specialised care; 0.48 for primary care). However, in this figure, we can also see that heterogeneity in specialised care treatment is higher than in primary care treatment. In fact, there are two studies [40,46] with outcomes of 3.18 and 2.49, respectively, which are the source of this difference. This higher heterogeneity in specialised care treatments compared with primary care treatments is also found in the remaining types of outcome.

In Table 8 we can see the influence of moderating variables on all of the outcomes assessed (overall efficacy), on the specific outcome Global function and on the Fibromyalgia Impact Questionnaire (FIQ). Obviously, we could have included other outcomes and questionnaires but owing to the great amount of information, we selected these variables because they seem to be the most used in assessing the efficacy of fibromyalgia treatments. The results when the other outcomes or questionnaires are analysed are quite similar.

**Table 8:** Influence of moderating variables on all the outcomes assessed, on the Fibromyalgia Impact Questionnaire and on the Global Function

	**All the outcome assessed**				**Fibromyalgia Impact Questionnaire**				**Global function**			
	Std dif in means	Lower	Upper	p-value	Std dif in means	Lower	Upper	p-value	Std dif in means	Lower	Upper	p-value

**Methodological quality**												
1 to 2	**1,21**	0.74	1.69	0.000					**1.40**	0.75	2.04	0.000
3 to 4	**0.57**	0.42	0.72	0.000	**0.66**	0.37	0.95	0.000	**0.58**	0.33	0.83	0.000
5 to 6	**0.23**	0.14	0.31	0.000	**0.36**	0.18	0.55	0.000	**0.37**	0.22	0.51	0.000
**Overall**	**0.33**	0.26	0.41	0.000	**0.45**	0.29	0.60	0.000	**0.46**	0.33	0.58	0.000

**Kind of treatment**												
Pharmacological	**0.42**	0.32	0.53	0.000	**0.59**	0.29	0.89	0.000	**0.54**	0.33	0.74	0.000
Non-pharmacological	**0.63**	0.43	0.83	0.000	**0.52**	0.26	0.79	0.000	**0.52**	0.26	0.79	0.000
**Overall**	**0.47**	0.37	0.56	0.000	**0.55**	0.35	0.75	0.000	**0.53**	0.37	0.70	0.000

**Length of treatment (weeks)**												
0 to 8	**0.73**	0.57	0.89	0.000	**0.83**	0.35	1.30	0.001	**0.82**	0.50	1.14	0.000
9 to16	**0.20**	0.11	0.28	0.000	**0.35**	0.20	0.49	0.000	**0.33**	0.20	0.46	0.000
17 to 24	**0.36**	0.14	0.58	0.001	**0.73**	0.29	1.16	0.001	**0.35**	-0.01	0.71	0.055
**Overall**	**0.31**	0.24	0.38	0.000	**0.42**	0.29	0.55	0.000	**0.40**	0.28	0.51	0.000

**Age**												
30 to 39	**1.41**	0.95	1.88	0.000	**1.41**	0.97	1.85	0.000	**1.41**	0.97	1.85	0.000
40 to 49	**0.44**	0.35	0.54	0.000	**0.37**	0.25	0.50	0.000	**0.39**	0.28	0.51	0.000
50 to 59	**0.50**	0.12	0.88	0.010	**0.99**	-0.22	2.20	0.108	**0.99**	-0.22	2.20	0.108
**Overall**	**0.48**	0.39	0.58	0.000	**0.46**	0.34	0.58	0.000	**0.47**	0.35	0.58	0.000

**Women (%)**												
**< 80**	**3.22**	0.68	5.76	0.013								
80 to 89	**0.70**	0.49	0.91	0.000	**0.96**	0.30	1.62	0.004	**0.85**	0.46	1.24	0.000
90 to 99	**0.33**	0.23	0.44	0.000	**0.37**	0.13	0.61	0.002	**0.31**	0.14	0.47	0.000
100	**0.40**	0.25	0.55	0.000	**0.50**	0.23	0.78	0.000	**0.50**	0.23	0.78	0.000
**Overall**	**0.41**	0.33	0.49	0.000	**0.46**	0.29	0.64	0.000	**0.42**	0.28	0.55	0.000

**Duration of disorder (years)**												
0 to 5	**0.85**	0.57	1.13	0.000	**0.57**	0.21	0.93	0.002	**0.66**	0.34	0.98	0.000
6 to10	**0.36**	0.27	0.45	0.000	**0.45**	0.26	0.64	0.000	**0.38**	0.22	0.54	0.000
11 to15	**0.16**	0.02	0.29	0.023								
**Overall**	**0.36**	0.29	0.43	0.000	**0.49**	0.33	0.65	0.000	**0.45**	0.31	0.58	0.000

**Country**												
Germany	**0.36**	0.17	0.56	0.000								
Belgium	**0.94**	0.73	1.15	0.000					**1.02**	0.35	1.70	0.003
Brazil	**1.13**	0.46	1.80	0.001	**0.99**	-0.22	2.20	0.108	**0.99**	-0.22	2.20	0.108
Canada	**0.35**	0.20	0.50	0.000	**0.38**	0.17	0.59	0.000	**0.29**	0.11	0.46	0.001
Finland	**0.10**	-0.08	0.28	0.283								
Netherlands	**0.09**	-0.21	0.39	0.567								
Norway	**0.30**	-0.11	0.71	0.157								
Sweden	**0.35**	0.05	0.64	0.022	**0.45**	0.03	0.88	0.038	**0.45**	0.03	0.88	0.038
Switzerland	**0.02**	-0.21	0.26	0.836								
Turkey	**2.19**	1.31	3.08	0.000	**1.41**	0.97	1.85	0.000	**1.41**	0.97	1.85	0.000
USA	**0.39**	0.26	0.51	0.000	**0.34**	0.16	0.53	0.000	**0.36**	0.21	0.50	0.000
**Overall**	**0.36**	0.30	0.43	0.000	**0.46**	0.33	0.58	0.000	**0.42**	0.31	0.52	0.000

In Table 8 we can also see that an improvement in the methodological quality of the studies is accompanied by a reduction in size effect in the Global Function outcome (the same is found for FIQ scores or for overall efficacy), owing to lesser heterogeneity. Type of treatment, whether pharmacological or non-pharmacological, did not modify the mean effect size in any of the three variables assessed.

On the contrary, shorter length of treatment favours differences that increase size effect. However, these differences can be explained by higher heterogeneity in the studies with shorter treatments, as can be seen on Figure 2 and not by a decrease in the therapeutic effect in longer treatments. With regard to mean participant age, we can observe higher improvement in all outcomes assessed in younger patients. However, the number of studies that evaluate the period of age extremes (young and older people) and assess overall efficacy is low. There are no differences in outcome in relation to gender in any of the three variables evaluated. Finally, the duration of the disorder influences the outcome: a shorter evolution of the disease is associated with higher improvement in any outcome. Again, the number of these kinds of studies is low and heterogeneity is greater, so interpretation of the results is more subjective.

Statistical heterogeneity has been assessed by inconsistence [63]; in our study this is 75%, which is considered to be highly inconsistent. In these cases, the use of random effects analysis is recommended, which we did. A funnel plot between standard error and mean standardised difference, a quality measure to assess publication bias, indicates that most studies are distributed around the central line and are placed in the middle of the graph. There are some small sample studies scattered on the right and on the lower part of the graph that imbalance the weight towards positive values.

## Discussion

There have been studies assessing multi-modal treatments in primary care [64] and trying to improve the efficacy of primary care treatments for patients with fibromyalgia through better communication [65]. However, to the best of our knowledge this is the first meta-analysis on the efficacy of the treatment of fibromyalgia according to level of care. The clinical and economical relevance of this disorder makes this a key question of research in free, universal health systems in which general practitioners are the gateway to the system. Prevalent and chronic disorders such as fibromyalgia are a huge cost to the health care system [3] and it is necessary to demonstrate whether treatment in a specialised care setting improves the outcome compared with its routine management in a primary care setting.

Only 33 studies from 594 papers examined met the inclusion criteria of our study. These 33 RCTs assessed 120 treatment interventions on patients diagnosed with primary fibromyalgia, 4505 (57.8%) of whom were allocated to primary care and 3284 (42.2%) to specialised care. The sample was made up of middle-aged women, with an average duration of the disorder of six to 10 years, mainly treated with pharmacological approaches. Most of the studies were carried out in the USA and Canada and were published after 2000. Owing to the great variety of outcomes and questionnaires used to assess the patients, we have summarised the results of the most frequently used in the studies revised: global function, pain and FIQ. The quality of the studies was rather low with only one-third of them rating 5+ on the Oxford Scale.

The studies by Yildiz and colleagues [40] and Edinger and colleagues [46] could be considered as "outliers" because the treatments assessed in both studies were much more efficacious than the other treatments allocated to specialised care, but the size sample in both studies was small and the duration of treatment somewhat short. However, we have not ruled out these two studies from the meta-analysis for the following reasons:

• These studies fulfil the stringent selection criteria of the meta-analysis. Methodological quality was rated independently and this variable is not an exclusion criteria.

• We expected this meta-analysis to show great heterogeneity owing to the different kinds of treatments included. We can not eliminate these studies merely as a result of their heterogeneity, since they are as valuable as the other studies included. We have used a random effects model for the analysis.

• Both studies assess non-pharmacological treatments and both were allocated to specialised care. To exclude them could bias the study towards pharmacological treatments and primary care.

• We recalculated the meta-analysis excluding these two studies and the results were the same: there were no significant differences in the efficacy of the treatments for fibromyalgia when comparing primary care and specialised care.

Our meta-analysis demonstrates that there are no differences in the overall outcome of fibromyalgia regardless of the level of care in which the patient is treated. This article only summarises some outcomes and questionnaires, but we have not found differences favouring either specialised or primary care for any of the seven outcomes or the many questionnaires assessed. In the case of quality of life, the two levels of care could not be compared. We consider that the external validity of these data is high because the selection criteria of the studies allow it to be generalised to most western health services. However, with respect to internal validity, this data should be analysed cautiously because statistical heterogeneity was important for specialised care studies whereas primary care studies show great homogeneity. In any case, the study points to moderate efficacy of any of the treatments described for fibromyalgia and similar efficacy in both primary and specialised levels of care.

Two of the variables that improve treatment efficacy in fibromyalgia are low quality of the studies and shorter duration of treatment, although both of these are biased by the heterogeneity of the studies. Other variables that also improve outcome, which are not biased by the heterogeneity of the studies, are younger patient age and shorter duration of the disorder. In elderly patients, treatment efficacy shows no significant difference when compared with the control group. However, gender and type of treatment (pharmacological vs. psychological) are not related to outcome. These results insist on the importance of an early diagnosis of the disorder, a fact that is usually related to a younger age of the patient.

Three are three main limitations to this research:

• Heterogeneity of the disorder: there are considered to be several subgroups of patients with fibromyalgia [66], so each of them could have different treatment of choice and, consequently, the results of this study could be different for every subgroup.

• The variability of outcomes used in fibromyalgia: it is difficult to obtain a single outcome summarising the efficacy of the treatment.

• Allocation of treatments to a level of care is a subjective matter: some treatments are new and we cannot foresee which level they will be used at, while others that can theoretically be used in primary care are scarcely utilised at this level. Decisions on which level these should be allocated to had been attained by agreement among four clinicians belonging to different levels and several specialities. Another criticism could be that all the treatments allocated to primary care could also be used at a specialist level, so the comparison should have been primary care treatments vs. all fibromyalgia treatments. However, we did not used this approach because our aim was to assess what was the added value in using specialised treatments for increasing efficacy of fibromyalgia treatment.

The health system operating in Spain is quite similar to the UK and Dutch health systems and is also comparable to other European and Western health systems, with some modifications. The Spanish health system is available to all Spaniards and citizens of the EU and is completely free at all levels. For this reason, there are no economic limitations to accessibility to the system in Spain, and only geographical limitations may exist (inhabitants of small and isolated mountain villages would have more difficulty accessing large hospitals). In this sense, the results of our study could be useful for most Western health systems. Obviously, the results of the meta-analysis are more difficult to extrapolate to countries such as the USA where primary care is not the entrance gate to the system. However, the most important conclusion that can be extrapolated from the study is that family doctors can see similar improvements in their patients with their treatment than specialised clinics in the management of patients with fibromyalgia.

## Conclusion

Based on this meta-analysis and despite the heterogeneity of specialised care studies, and of the other limitations described, there is no clear indicator for treating fibromyalgia in specialised care or for the development of specialised units for the treatment of this disorder. According to this study, the same moderate efficacy can be obtained in primary care settings with the routine treatments available at this level. Obviously, any new treatment could upset the balance of this comparison and, whatever the case, new research studies that are more focused on cost-efficacy analysis are necessary to confirm this data.

## Abbreviations

ACR = American College of Rheumatology; CI = confidence interval; FIQ = fibromyalgia impact questionnaire; RCT = randomized controlled trial; SDM = standardised differences in means.

## Competing interests

The authors declare that they have no competing interests.

## Authors' contributions

JGC is the principal researcher and developed the original idea for the study. The study design was further developed by JM and RM. EFG and MS carried out the electronic search and reviewed the studies. EA and JM developed the statistical methods. All authors have read and corrected draft versions and approved the final version.

## Supplementary Material

Additional file 1 A file containing the searching strategy used to select the papers included in the study. Click here for file
