# Supplementary material for: A meta-analysis of the efficacy of fibromyalgia treatment according to level of care
Source: Arthritis Res Ther. 2008 Jul 15;10(4):R81. doi: 10.1186/ar2455 (PMC2575627; doi:10.1186/ar2455)
Supplement: Additional file 1 — A file containing the searching strategy used to select the papers included in the study. [file ar2455-S1.doc]

Annex I. Searching strategy

Searching in PUBMED:

**#1** (("Fibromyalgia/diet therapy"[MeSH] OR "Fibromyalgia/drug therapy"[MeSH] OR "Fibromyalgia/psychology"[MeSH] OR "Fibromyalgia/radiotherapy"[MeSH] OR "Fibromyalgia/rehabilitation"[MeSH] OR "Fibromyalgia/therapy"[MeSH]) OR (fibromyalgia[ti] AND (treatment[ti] OR therapeutics[ti] OR therapy[ti] OR (psic[ti] OR psicain[ti] OR psicaine[ti] OR psichiatria[ti] OR psichiatrici[ti] OR psichiatrico[ti] OR psichica[ti] OR psichicae[ti] OR psichodidae[ti] OR psichosomatic[ti] OR psickle[ti] OR psico[ti] OR psicoanalisi[ti] OR psicoanalisis[ti] OR psicoanalitico[ti] OR psicofuranine[ti] OR psicofuranosyl[ti] OR psicofuranosylpurine[ti] OR psicoline[ti] OR psicologia[ti] OR psicologiche[ti] OR psicopedagogico[ti] OR psicoperidol[ti] OR psicoprofilassi[ti] OR psicopyranose[ti] OR psicosamines[ti] OR psicose[ti] OR psicoselysine[ti] OR psicosocial[ti] OR psicosociologia[ti] OR psicosoma[ti] OR psicosomatica[ti] OR psicost[ti] OR psicosuria[ti] OR psicotard[ti] OR psicotecnico[ti] OR psicsi[ti]))))

**#2** (systematic review [ti] OR meta-analysis [pt] OR meta-analysis [ti] OR systematic literature review [ti] OR consensus development conference [pt] OR practice guideline [pt] OR cochrane database syst rev OR acp journal club OR health technol assess OR evid rep technol assess summ)  OR  ((evidence based[ti] OR evidence-based medicine [mh] OR best practice* [ti] OR evidence synthesis [tiab]) AND (review [pt] OR diseases category[mh] OR behavior and behavior mechanisms [mh] OR therapeutics [mh] OR evaluation studies[pt] OR validation studies[pt] OR guideline [pt])) OR
((systematic [tw] OR systematically OR critical [tiab] OR (study selection [tw]) OR (predetermined OR inclusion AND criteri*) OR exclusion criteri* OR "main outcome measures" OR "standard of care" OR "standards of care") AND  (survey [tiab] OR surveys [tiab] OR overview* OR review [tiab] OR reviews [tiab] OR search* OR handsearch OR analysis [tiab] OR critique [tiab] OR appraisal OR  (reduction AND risk AND (death OR recurrence))) AND (literature [tiab] OR articles [tiab] OR publications [tiab] OR publication [tiab] OR bibliography [tiab] OR bibliographies [tiab] OR published [tiab] OR unpublished OR citation OR citations OR database [tiab] OR internet [tiab] OR textbooks [tiab] OR references OR scales [tw] OR papers [tw] OR datasets OR trials [tiab] OR meta-analy* [tw] OR (clinical [tiab] AND studies [tiab]) OR treatment outcome)) NOT  (case report [ti] OR letter [pt] OR newspaper article [pt] OR comment [pt])

**#3** #1 AND #2

Searching in EMBASE_

**'fibromyalgia'**/exp/dm_dt,dm_rt,dm_rh,dm_th AND ([english]/lim OR [french]/lim OR [german]/lim OR [italian]/lim OR [portuguese]/lim OR [spanish]/lim) AND ([adult]/lim OR [aged]/lim) AND [embase]/lim AND [1990-2006]/py AND ([cochrane review]/lim OR [meta analysis]/lim OR [systematic review]/lim) AND [embase]/lim AND [1990-2006]/py

Searching in Cochrane:

**[#1](http://212.188.234.56/newgenClibPlus/ASP/History.asp?updhist=1#%23)**(fibromyalgia or fibromialgia)

**[#2](http://212.188.234.56/newgenClibPlus/ASP/History.asp?updhist=1#%23)** FIBROMYALGIA [dh:dt:px:th:rh:rt] expandir todos los árboles (MeSH)

**[#3](http://212.188.234.56/newgenClibPlus/ASP/History.asp?updhist=1#%23)** (#1 or #2)

Searching in PsychInfo

1.- fibromyalgia [kw] and (therapy or

treatment or rehabilitation)

2.- Systematic Review OR

Meta Analysis

3.- 1 AND 2
